# The Clinical Utility of Point-of-Care Tests for Influenza in Ambulatory Care: A Systematic Review and Meta-analysis

**DOI:** 10.1093/cid/ciy837

**Published:** 2018-10-04

**Authors:** Joseph Jonathan Lee, Jan Y Verbakel, Clare Rosemary Goyder, Thanusha Ananthakumar, Pui San Tan, Phillip James Turner, Gail Hayward, Ann Van den Bruel

**Affiliations:** 1 Nuffield Department of Primary Care Health Sciences, University of Oxford, United Kingdom; 2 Academic Center for General Practice, Katholieke Universiteit Leuven, Belgium

**Keywords:** influenza, diagnostics

## Abstract

**Background:**

Point-of-care tests (POCTs) for influenza are diagnostically superior to clinical diagnosis, but their impact on patient outcomes is unclear.

**Methods:**

A systematic review of influenza POCTs versus usual care in ambulatory care settings. Studies were identified by searching six databases and assessed using the Cochrane risk of bias tool. Estimates of risk ratios (RR), standardised mean differences, 95% confidence intervals and I2 were obtained by random effects meta-analyses. We explored heterogeneity with sensitivity analyses and meta-regression.

**Results:**

12,928 citations were screened. Seven randomized studies (n = 4,324) and six non-randomized studies (n = 4,774) were included. Most evidence came from paediatric emergency departments. Risk of bias was moderate in randomized studies and higher in non-randomized studies. In randomized trials, POCTs had no effect on admissions (RR 0.93, 95% CI 0.61–1.42, I2 = 34%), returning for care (RR 1.00 95% CI = 0.77–1.29, I2 = 7%), or antibiotic prescribing (RR 0.97, 95% CI 0.82–1.15, I2 = 70%), but increased prescribing of antivirals (RR 2.65, 95% CI 1.95–3.60; I2 = 0%). Further testing was reduced for full blood counts (FBC) (RR 0.80, 95% CI 0.69–0.92 I2 = 0%), blood cultures (RR 0.82, 95% CI 0.68–0.99; I2 = 0%) and chest radiography (RR 0.81, 95% CI 0.68–0.96; I2 = 32%), but not urinalysis (RR 0.91, 95% CI 0.78–w1.07; I2 = 20%). Time in the emergency department was not changed. Fewer non-randomized studies reported these outcomes, with some findings reversed or attenuated (fewer antibiotic prescriptions and less urinalysis in tested patients).

**Conclusions:**

Point-of-care testing for influenza influences prescribing and testing decisions, particularly for children in emergency departments. Observational evidence shows challenges for real-world implementation.

## Teaser

We reviewed the evidence for the impact of point-of-care diagnostic tests for influenza. Testing reduced further investigation with chest radiography and full blood counts, and increased antiviral prescribing, but had no impact on antibiotic use, returning for care, or admissions.

Influenza is a major global disease. The World Health Organization estimates 1 billion infections and half a million deaths from respiratory complications each year [1, 2]. Influenza affects healthcare, society, and the world economy, although often the impact is attributed to other infections such as pneumonia [3–5]. In the United Kingdom, influenza is responsible for more than half a million primary care consultations and more than 19000 hospital admissions and deaths each year, though they are often not recognized as influenza [5].

Many respiratory infections cause the same syndrome as influenza; these are referred to as influenza-like illnesses (ILIs) [1]. Despite being unable to distinguish clinical features of influenza from other causes of ILI, clinical diagnosis is widespread [6, 7]. Diagnostic uncertainty in ILI contributes to antibiotic prescribing [8], so diagnostics could improve antimicrobial stewardship. UK guidance from the National Institute for Health and Care Excellence recommends no antibiotic prescribing for patients with respiratory tract infections that are likely to be self-limiting, including influenza, unless patients are systemically unwell or at higher risk of unfavorable outcomes [9]. Nonetheless, 14%–40% of patients with influenza are prescribed antibiotics [10, 11].

Influenza point-of-care tests (POCTs) are specific (>98%), but rapid antigen detection tests (RADTs) have low sensitivity compared to nucleic acid amplification tests (53%–54% vs 92%–95%) [12]. Even RADTs offer more accurate diagnoses than clinical evaluation and are fast enough to influence prescribing in ambulatory settings [6, 13]. We cannot assume POCTs will automatically lead to beneficial outcomes [14]. This review aims to collate the available evidence on the impact of point-of-care influenza tests in ambulatory care. We sought to examine clinically relevant impacts, including hospital admissions, antibiotic and antiviral prescribing, and the use of other diagnostic tests.

## METHODS

We published the study protocol prospectively [15]. The search strategy for this review targeted all controlled studies that evaluated the clinical impact of any POCTs in ambulatory care in the 6 most important medical databases (Supplementary Materials). We updated the search, which included terms for POCTs for any condition, on 21 March 2017. We selected studies of influenza POCTs at the full text stage, and will publish findings for other POCTs elsewhere.

### Inclusions and Exclusions

Participant demographics and preexisting conditions were not restricted. We included the following ambulatory care settings: primary care, emergency department, and clinic, but we did not include studies of hospitalized patients. We excluded tests sent to a different location for analysis, such as a laboratory. We included any POCTs for diagnosis of influenza, with or without other tests. Nondiagnostic biomarkers alone were ineligible. We compared POCTs with usual care. This could include no testing or laboratory tests for influenza, but not another novel test. We included all quantitative clinical outcomes, excluding health economic outcomes. When extracting data on further tests, we grouped routine blood tests with full blood counts and combined urinalysis techniques. We included randomized, controlled trials (RCTs) and nonrandomized studies for separate analysis. We excluded study designs that precluded comparisons between tested and untested participants (case studies, case series, and studies without controls).

We screened articles independently in duplicate at title, abstract, and full-text levels. Discussion or a third reviewer resolved conflicts. J. L. extracted data and assessed quality, and J. V. checked data extraction and quality assessment. We contacted corresponding authors for unpublished information.

### Analyses

We used random effects meta-analyses to generate pooled estimates with 95% confidence intervals (CIs) and I^2^. We estimated risk ratios (RRs) for dichotomous outcomes and mean differences or standardized mean differences (where outcomes may have been measured differently) for continuous outcomes. We planned to calculate missing estimates using methods from the Cochrane handbook [16] (but were unable to do so) and used sensitivity analyses, omitting studies to explore heterogeneity. We used post hoc random effects metaregression to explore heterogeneity attributable to the prevalence of influenza and baseline outcomes where 10 or more studies reported an outcome using the log odds scale to allow linear regression [17]. We used Covidence software for citation management [18]. Metaanalysis was undertaken with Revman 5.3 [19], metaregression with Stata 14 SE [20].

## RESULTS

**Figure 1.**
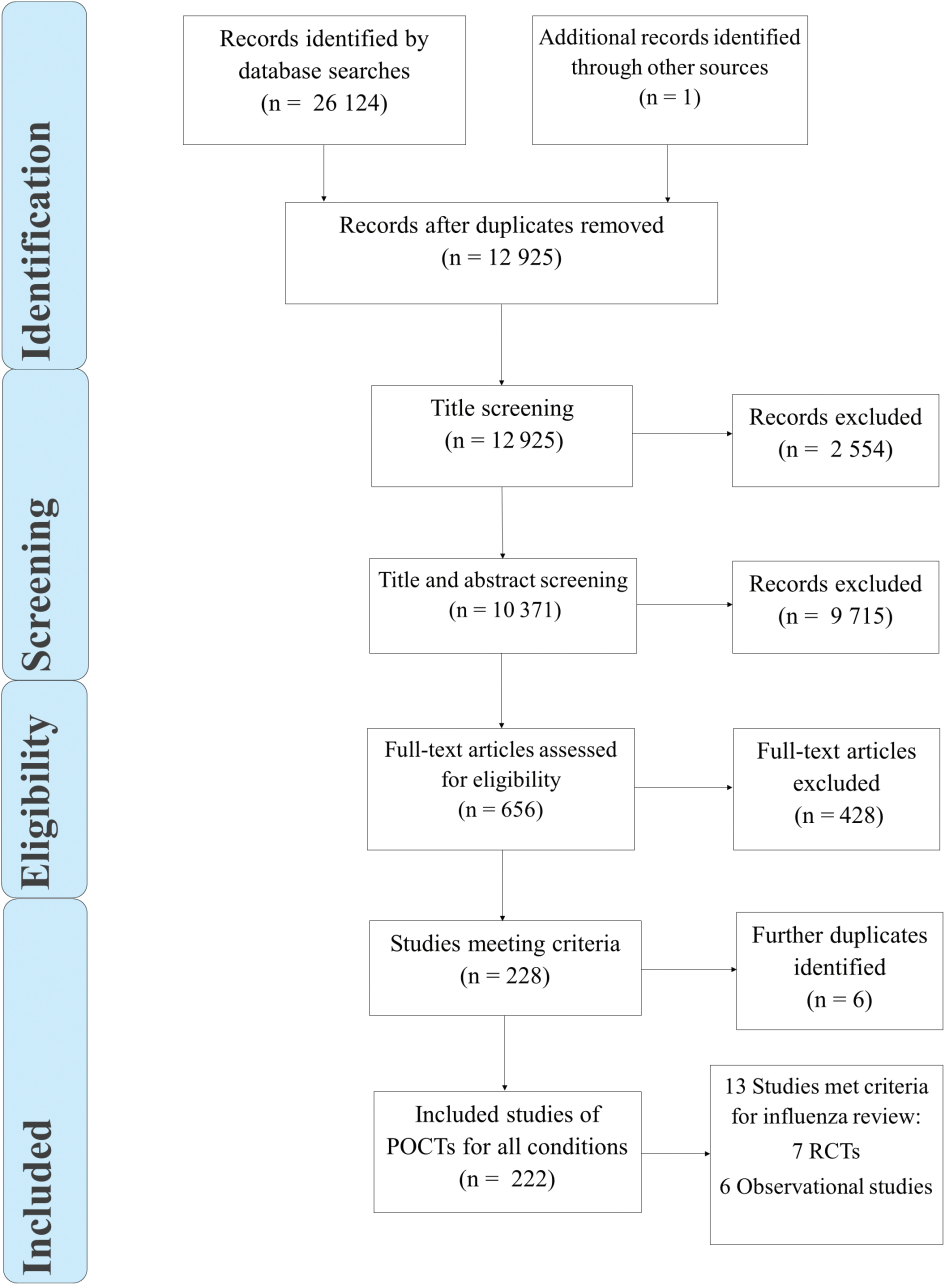
PRISMA flowchart of included and excluded papers. Abbreviations: POCTs, point-of-care tests; PRISMA, Preferred Reporting Items for Systematic Reviews and Meta-Analyses; RCT, randomized, controlled trials.

The searches resulted in 12928 unique records (Figure 1); 12269 were excluded by title and abstract screening, and the remaining 659 underwent full-text review. A total of 225 full texts were eligible for inclusion in 1 or more review. Thirteen studies were of influenza POCTs (Figure 1 and Table 1).

**Table 1.** Characteristics of Included Studies

Study	Design	Number of Participants	Age Range	Setting	Influenza Prevalence, %	Point-of-Care Tests	Comparator
Abanses et al 2006 [21]	RCT–24-hour time blocks randomized	1007	3–36 months	ED, United States	28.1	Directigen Flu A + B	No test
Bonner et al 2003 [22]	RCT	418	2 months–21 years^a^	ED, United States	49.7	FluOIA	Tested, but results not disclosed
Cohen et al 2007 [23]	Cluster RCT	602	0.7–17 years	ED, France	54.0	Quickvue	No test
Doan et al 2009 [24]	RCT	204	3–36 months	ED, Canada	21.1	Direct immunofluorescence panel	No test unless ordered later
Esposito et al 2003 [25]	RCT	957	0–15 years	ED, Italy	9.0	Quickvue	No test
Iyer et al 2006 [26]	Quasi RCT	700	2–24 months	ED, United States	30.4	Quickvue	“Standard test” undertaken after discharge
Poehling et al 2006 [27]	RCT	468	0–5 years	ED/acute care clinic, United States	24.9	Quickvue	Culture and polymerase chain reaction
Jeong et al 2014 [28]	Retrospective record review	437	All ages	ED, Korea	33.8	SD Bioline Influenza Antigen Test	Period before implementation
Jun et al 2016 [29]	Retrospective record review	474	All ages	ED, Korea	11.5	Unclear, “rapid antigen test”	Period before implementation
Lacroix et al 2015 [30]	Comparison of decisions pre- and post-results	340	1 month–5 years	ED, France	47.1	Quickvue	Decision before result revealed
Nitsch-Osuch et al 2013 [31]	Open, nonrandomized comparison	256	0–5 years	PC, Poland	30.4	BD Directigen EZ FluA + B	No test
Özkaya et al 2009 [32]	Single blinded comparison	97	3–14 years	ED, Turkey	32.0	Unclear, “Influenza A ⁄ B Rapid Test”	No test
Theocharis et al 2010 [33]	Retrospective record review	3412	All ages	PC home visits, Greece	49.2	Influ A&B Uni-Strip—Dry Swabs (C-1512)	No test

Abbreviations: ED, emergency department; PC, primary care; RCT, randomized controlled trial.

^a^Most aged <36 months.

### Characteristics of Included Studies

There were 7 randomized trials [21–27], including 1 quasi-randomized and 1 cluster randomized trial ([26] and [23]; Table 1). Usual care varied. Four trials used no test [21, 23–25], 2 used laboratory-based influenza tests [26, 27], and 1 used the POCT in the comparator group but concealed the result [22]. The remaining 6 studies were not randomized studies [28–33]. Two compared records before and after the introduction of POCTs to Korean emergency departments [28, 29], and 1 compared tested and untested patients in Greek primary care home visit records [33]. Three nonrandomized studies were more experimental, 1 compared what clinicians said their clinical decisions would be before and after revealing the test results [30], 1 was a single-blinded trial in which allocation was not clearly randomized [32], and 1 was a prospective open cohort [31].

Six randomized trials [21, 22, 24–27] were conducted in emergency departments, and 1 cluster RCT was performed in a primary care setting [23] (Table 1). Four nonrandomized studies were in emergency departments [28–30, 32], and 2 were in primary care settings [31, 33]. The age of participants varied, but pediatric populations were dominant, and most evidence comes from children aged <5 years. All randomized trials and 3 nonrandomized studies were in children. Three nonrandomized studies included adults and children [28, 29, 33], 2 included children aged <5 years [30, 31], and 1 included children aged 3 to 14 years [32] (Table 1).

Six trials used rapid antigen detection kits [21, 34], of which 4 [23, 25–27] used the Quickvue Influenza A&B by Quidel. One trial used a panel test, a direct immunofluorescence assay that targets adenovirus, respiratory syncytial virus, parainfluenza, and influenza [24] (Table 1). All 6 nonrandomized studies used rapid antigen detection kits, 1 of which was Quickvue Influenza A&B [30].

**Figure 2.**
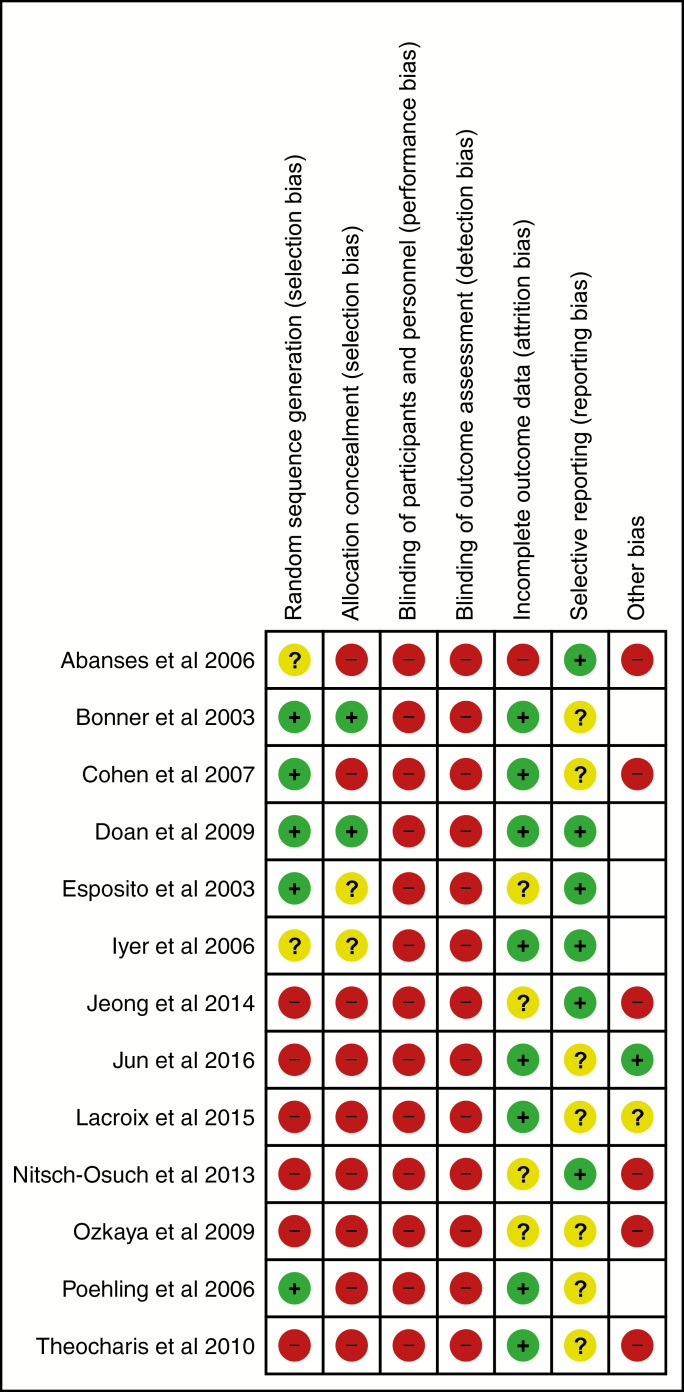
Risk of bias summary for included studies.

Randomized studies were of moderate risk of bias; nonrandomized studies had a higher risk (Figure 2). None of the studies were able to blind participants and personnel to testing or test results. We found no study that blinded outcome assessors to test status.

### Patient Outcomes

No study reported mortality and morbidity measures, such as illness course. Most outcomes were measures of impact on management decisions and further investigation (Table 2).

**Table 2.** Summary of Pooled Results

	Randomized Trials		Nonrandomized Studies	
Outcome	Studies (n)^a^	Pooled Effect Estimate	Studies (n) ^a^	Pooled Effect Estimate
Admission to hospital	2 (1657)	RR, 0.93; 95% CI, 0.61 to 1.42; I^2^ 34%	2 (3739)	RR, 0.73; 95% CI, 0.49 to 1.09; I^2^ 0%
Returning for care	2 (899)	RR, 1.00; 95% CI, 0.77 to 1.29; I^2^ 7%	...	...
Time in emergency department	3 (1826)	SMD, –0.03; 95% CI, –0.14 to 0.07; I^2^ 12%	2 (891)	SMD, 0.49; 95% CI, –0.15 to 1.14; I^2^ 96%
Antibiotic prescribing	7 (4324)	RR, 0.97; 95% CI, 0.82 to 1.15; I^2^ 70%	5 (4602)	RR, 0.64 95% CI, 0.48 to 0.86; I^2^ 81%
Antibiotics duration, days	1 (592)	MD, 0.00; 95% CI, to 0.35 to 0.35	...	...
Antiviral prescribing	3 (1461)	RR, 2.65; 95% CI, 1.95 to 3.60; I^2^ 0%	3 (3995)	RR, 11.36; 95% CI, 0.82 to 157.12; I^2^ 88%
Any further testing	1 (468)	RR, 0.83; 95% CI, 0.65 to 1.07	1 (340)	RR, 0.53; 95% CI, 0.43 to 0.64
Routine blood work or full blood count	7 (4161)	RR, 0.80 95% CI, 0.69 to 0.92; I^2^ 0%	2 (669)	RR, 0.85; 95% CI, 0.18 to 4.1; I^2^ 92%
Blood cultures	3 (2098)	RR, 0.82; 95% CI, 0.68 to 0.99; I^2^ 0%	...	...
Chest radiography	7 (4161)	RR, 0.81; 95% CI, 0.68 to 0.96; I^2^ 32%	3 (1009)	RR, 0.77; 95% CI, 0.57 to 1.05; I^2^ 65%
Urinalysis	5 (2742)	RR, 0.91; 95% CI, 0.78 to 1.07 I^2^ 20%	1 (340)	RR, 0.47; 95% CI, 0.37 to 0.61
Lumbar punctures	3 (2098)	RR, 1.07; 95% CI, 0.45 to 2.54; I^2^ 0%	...	...
Respiratory syncytial virus testing	1 (1007)	RR, 0.40, 95% CI, 0.26 to 0.63	...	...

Pooled results compare point-of-care influenza testing with usual care, meta-analyzed with Mantel–Haenszel random effects models.

Abbreviations: CI, confidence interval; MD, mean difference; RR, relative risk; SMD standardised mean difference.

^a^ Studies indicates the number of included studies reporting outcome. n indicates total number of participants

### Management Decisions

Admissions to hospital were not reduced in any individual study or pooled estimates of randomized [25, 26] (RR, 0.93; 95% CI, 0.61 to 1.42; I^2^ = 34%) or nonrandomized studies [30, 33] (RR, 0.73; 95% CI, 0.49 to 1.09; I^2^ = 0%) (Supplementary Figure S1).

Patients returning for follow-up was an outcome in 2 randomized studies [24, 26] (Supplementary Figure S2). Neither suggested an effect of testing on returning for care nor did the pooled estimate (RR, 1.00; 95% CI, 0.77 to 1.29; I^2^ = 7%).

Time patients spent in emergency departments was unchanged by POCTs (Supplementary Figure S3). We pooled 3 randomized studies [21, 24, 26] (n = 1826; standardized mean difference −0.03; 95% CI, −0.14 to +0.07; I^2^ = 12%) and 2 nonrandomized studies [28, 29] (n = 891; standardized mean difference, 0.49; 95% CI, −0.15 to +1.14; I^2^ = 96%).

### Prescribing

**Figure 3.**
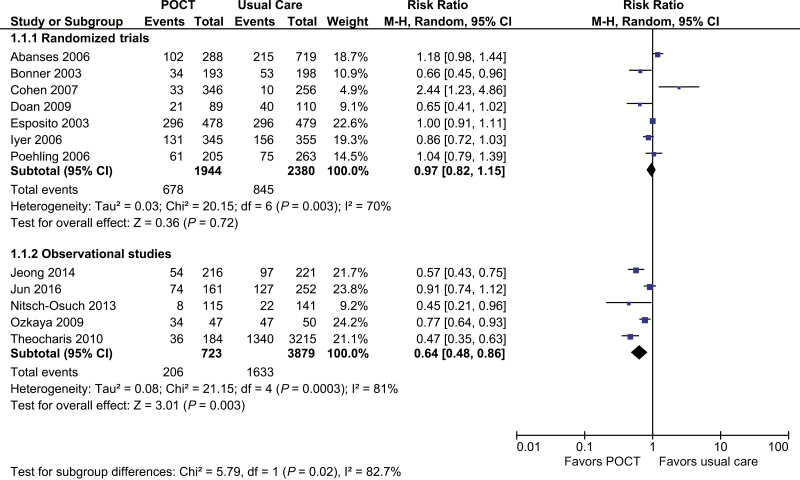
Antibiotic prescribing. Abbreviations: CI, confidence interval; POCT, point-of-care test; RCT, randomized, controlled trial.

Antibiotic prescribing was the most common outcome, reported in 7 randomized [21–27] and 5 nonrandomized studies [28, 29, 31–33]. RCTs showed no effect on antibiotic prescribing (RR, 0.97; 95% CI, 0.82 to 1.15; I^2^ = 70%; Figure 3). Of the 7 RCTs, 5 estimated no effect [21, 24–27], 1 a statistically significant decrease [22], and 1 a significant increase [23]. The Cohen et al study is the one cluster RCT and did not account for clustering in analysis or give sufficient information to estimate accurate standard errors. We therefore performed sensitivity analyses by removing this study [23]. The pooled estimate was robust to removing the Cohen et al study, and heterogeneity was lowered (RR, 0.94; 95% CI, 0.81 to 1.08; I^2^ = 63%). In a second sensitivity analysis using data from 3 randomized trials (n = 1559) [22, 26, 27], we compared antibiotic prescribing in patients who tested positive for influenza with patients who tested negative (Supplementary Figure S4). There was no significant effect on prescribing in those with influenza (RR, 0.63; 95% CI, 0.32 to 1.22; I^2^ = 64%) and no evidence of an increase in antibiotic prescribing in patients without influenza (RR, 1.02; 95% CI, 0.85 to 1.23; I^2^ = 0%).

Of the 5 nonrandomized studies that reported on antibiotic prescribing, 4 [28, 31–33] reported significant reductions. Meta-analysis showed a strong association between POCTs and reduced antibiotic prescribing but with strong evidence of statistical heterogeneity (RR, 0.64; 95% CI, 0.48 to 0.86; I^2^ = 81%). Random effects metaregression of all study types showed much of the heterogeneity in study log odds ratios could be attributed to the baseline proportion of patients with influenza and antibiotic prescribing (antibiotic prevalence in control arm × influenza prevalence; Supplementary Figure S5). The proportion of variation in antibiotic prescribing between studies (I^2^) that the model attributed to this feature (adjusted R^2^) was 79% (*P* = .003).

A single randomized study estimated the duration of antibiotic treatment. Esposito et al [23] found no evidence of a difference between groups (mean difference, 0.00; 95% CI, −0.35 to 0.35).

Prescribing of antivirals was reported in 6 studies (n = 5056), 3 randomized [22, 23, 27] and 3 nonrandomized [30, 31, 33]. Meta-analysis of randomized studies showed an increase in antiviral prescribing with POCT use (RR, 2.65; 95% CI, 1.95 to 3.60; I^2^ = 0%; Supplementary Figure S6). When we excluded the Cohen at al study [23], borderline evidence of an effect remained (RR, 2.12; 95% CI, 1.00 to 4.51; I^2^ = 0%). Meta-analysis of nonrandomized studies showed no difference in antiviral prescribing and high heterogeneity (RR, 11.36; 95% CI, 0.82 to 157.12; I^2^ = 88%).

### Test Use

Ten studies reported the impact of influenza POCTs on additional tests (all 7 randomized trials and 3 nonrandomized studies [29–31]).

The composite outcome of any further testing was extractable from 2 studies (Supplementary Figure S7). A randomized trial estimated an RR of 0.83 (95% CI, 0.65 to 1.07) [27], and a nonrandomized study of decisions before and after test results were revealed to clinicians estimated an RR of 0.53 (95% CI, 0.43 to 0.64) [30].

**Figure 4.**
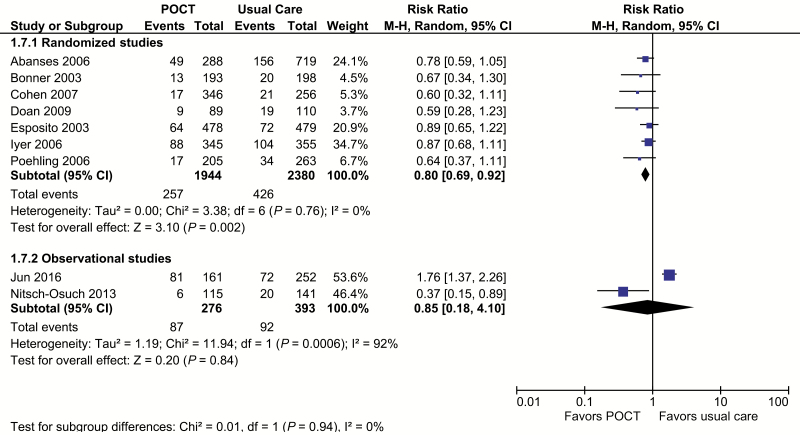
Routine bloods or full blood count. Forest plot of meta-analyses of randomized and observational studies reporting full blood counts or routine bloods comparing POCT vs usual care. Abbreviations: CI, confidence interval; POCT, point-of-care test; RCT, randomized, controlled trial.

Based on 7 randomized trials [21–27] including 4161 patients, POCTs reduce the use of routine blood tests by 20% (RR, 0.80; 95% CI, 0.69 to 0.92; I^2^ = 0%; Figure 4). None of these studies estimated significant effects on their own, but all had point estimates favoring POCTs. When we removed the Cohen et al study from a sensitivity analysis, the results were robust. The pooled result of the 2 nonrandomized studies [29, 31] (n = 669) showed a nonsignificant result (RR, 0.85; 95% CI, 0.18 to 4.1; I^2^ = 92%; Figure 4).

Three RCTs reported blood cultures [21, 22, 26]. All 3 had point estimates in the direction of a reduction, but none were significant. The pooled estimate showed a significant reduction in blood cultures with point-of-care testing (RR, 0.82; 95% CI, 0.68 to 0.99; I^2^ = 0%; Supplementary Figure S8).

**Figure 5.**
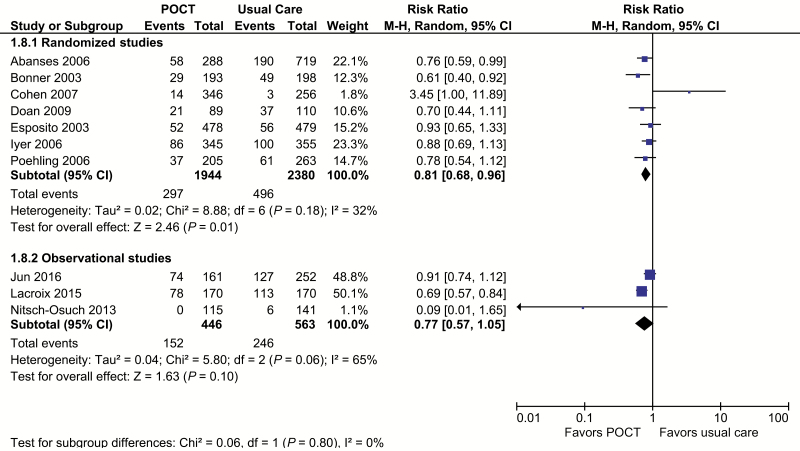
Chest radiography. Forest plot of meta-analyses of randomized and observational studies reporting chest radiography comparing POCT vs usual care. Abbreviations: CI, confidence interval; POCT, point-of-care test; RCT, randomized, controlled trial.

Chest radiography was reported in the 7 RCTs [21–27] and 3 nonrandomized studies [29–31]. Metaanalysis of the randomized trials (n = 4161) gave a pooled RR of 0.81 (95% CI, 0.68 to 0.96; I^2^ = 32%; Figure 5). The results from the Cohen et al study were in the opposite direction of the other studies; a sensitivity analysis without the Cohen et al study removed all heterogeneity, and the result was robust (RR, 0.80; 95% CI, 0.70 to 0.91; I^2^ = 0%). The 3 nonrandomized studies of 1009 participants had a pooled estimate that was similar to those of the randomized studies, but it was not significant (RR, 0.77; 95% CI, 0.57 to 1.05; I^2^ = 65%). The largest reductions tended to be in studies with higher influenza and higher chest radiography. Random effects metaregression included randomized and nonrandomized studies (Supplementary Figure S9). We regressed log odds ratios for chest radiography against the proportion of patients with influenza who might undergo chest radiography (influenza prevalence × radiography use in control arms). Up to 100% of between-study variance could be attributed to this combination of influenza prevalence and baseline requesting rates in studies of all study designs (adjusted R^2^ = 100%; *P* = .03).

Urinalysis was not affected by influenza point-of-care testing, based on the meta-analysis of 5 randomized studies (RR, 0.91; 95% CI, 0.78 to 1.07; I^2^ = 20%; Supplementary Figure S10) [21, 22, 24, 26, 27]. The only nonrandomized study looked at theoretical clinical decisions and found that urinalysis declined by approximately half in children with pyrexia of unknown origin (RR, 0.47; 95% CI, 0.37 to 0.61; Supplementary Figure S10) [30].

We found no evidence of an impact on lumbar punctures. This outcome was rare, 21 events in 3 randomized studies [21, 22, 26] of 2098 participants (RR, 1.07; 95% CI, 0.45 to 2.54; I^2^ = 0%; Supplementary Figure S11).

Respiratory syncytial virus testing was an outcome in only 1 influenza testing study. Abanses et al [21] reported evidence of a reduction (RR, 0.40; 95% CI, 0.26 to 0.63; Supplementary Figure S12).

## DISCUSSION

POCTs reduced the risk of routine blood tests by 20%, blood cultures by 18%, and chest radiography by 19% in RCTs. These results suggest POCTs have a role in reducing diagnostic uncertainty for children with ILI, but the impact on patient outcomes remains unclear. Antibiotic prescribing was not affected by testing, but prescriptions for antiviral medications more than doubled. POCTs did not affect time in the emergency department or numbers of patients returning for care. Most evidence came from RADTs, which are known to be specific but have low sensitivity [6]. Newer tests have higher sensitivity [12], which may increase their impact.

Nonrandomized studies had different results compared to RCTs that included reduced antibiotic prescribing, overall testing, and urinalysis. Routine blood tests were reduced in trials but not in nonrandomized studies. We attribute the differences to baseline prescribing rates and influenza prevalence but also to higher risk of bias. Diagnostics are complex interventions [35]; clinical context, flow, and timing are important components that affect impact. A POCT cannot reduce further testing unless the POCT result is available and considered before further tests are requested, which may not happen outside of trials. The nonrandomized result was driven by a large cohort study that included adults and children before and after a Korean emergency department introduced a POCT [29]. In that study, POCTs had become routine, so clinicians may have requested them at the same time as blood tests. Overall, tests and urinalysis were examined in only 1 nonrandomized study of questionable risk of bias [30]. Investigators asked clinicians for their decisions before and after having a result revealed to them. Asking in this way likely focused the clinicians’ attention on what they can do differently. The impact of POCTs may be less without this interaction, although a carefully designed implementation might replicate it.

The diagnostic accuracy of POCTs for influenza has been examined extensively in individual studies and systematic reviews [6, 12, 36], but we looked at direct evidence of clinical outcomes. We believe this is the first systematic review of the impact of influenza POCTs on clinical outcomes and includes all relevant primary studies. Individual studies had insufficient power to show effects. Pooling results from nonsignificant studies allowed us to reveal previously unknown effects on the outcomes of chest radiography, antiviral prescribing, blood cultures, and routine blood tests.

This review used a comprehensive search strategy and included a variety of study types. It is unlikely that we missed a large body of work that would change the interpretation of the results. Our inclusion of nonrandomized studies has advantages—a priori it was unlikely trials would be powered to address rarer and more serious complications of influenza, and this is what we found. Unfortunately, no observational evidence for these outcomes exists.

The available evidence limits this review. There is little evidence from primary care settings. Most of the evidence comes from low-sensitivity RADT tests. Higher-sensitivity tests would detect more influenza and increase prevalence estimates. In addition to the direct impact of fewer false negatives, better tests might increase clinicians’ confidence to act on results.

RCTs had lower risk of bias than nonrandomized studies. RCTs were at moderate risk, but future studies are unlikely to be much lower risk. The Cochrane risk-of-bias tool gives harsh results for trials of diagnostic tests as interventions. Allocation concealment is impossible because effects work through knowledge of the test result. Blinding of outcome assessment is also difficult for self-reported outcomes.

Our examination of between-study heterogeneity underlines the importance of the prevalence of both influenza and outcomes of interest to clinicians, future studies, and policy makers.

Studies of POCTs in both adults and children are needed; there is an evidence gap in primary care settings. Most patients are seen in primary care settings, but the low prevalence of serious outcomes would require large studies [37]. Studies will need to be even larger to account for influenza’s variable and generally low prevalence, even during epidemics. Consequently, there is a space for well-conducted observational studies.

Future studies should examine clinical course, mortality, and morbidity measures. They should report outcomes by POCT results as well as status because effects may differ by result. Studies should examine the results of additional tests as a proxy for appropriateness of further investigation [38]. Reducing negative tests implies efficiency, but reducing positive tests implies missed bacterial infections.

Future research should explore appropriate contexts for POCT use and implementation. Combinations of newer influenza tests with other POCTs, C-reactive protein, for example, may help better identify patients with bacterial coinfections and give clinicians confidence to conserve antibiotics. Bias assessment for randomized trials of tests as interventions and therefore idealized study designs and reporting guidelines should be a research priority.

POCTs for influenza have a role for children with ILIs, particularly in emergency departments and during influenza epidemics. There is little evidence for or against implementation in primary care. Clinicians should consider local practice before implementation. Influenza POCTs reduce blood tests and chest radiography, but the reduction is greatest in settings with high levels of additional tests.

Tests are not a substitute for clinical assessment. We have not addressed the appropriateness of reducing chest radiography, blood cultures, or routine blood tests. However, the vast majority of childhood infections are self-limiting illnesses, so reductions are likely to be appropriate. The benefit of antiviral prescribing is debatable. Recent reviews have suggested benefit [39], but a Cochrane review was derisive about the evidence for effectiveness [40].

## CONCLUSIONS

There is evidence from randomized trials that influenza POCTs influence clinical decisions in ambulatory care, resulting in fewer blood tests and chest radiographs. The evidence is mostly for rapid antigen tests in children in emergency department settings.

## Supplementary Data

Supplementary materials are available at *Clinical Infectious Diseases* online. Consisting of data provided by the authors to benefit the reader, the posted materials are not copyedited and are the sole responsibility of the authors, so questions or comments should be addressed to the corresponding author.

## Supplementary Material

ciy837_suppl_Supplementary_Figure_S1

ciy837_suppl_Supplementary_Figure_S2

ciy837_suppl_Supplementary_Figure_S3

ciy837_suppl_Supplementary_Figure_S4

ciy837_suppl_Supplementary_Figure_S5

ciy837_suppl_Supplementary_Figure_S6

ciy837_suppl_Supplementary_Figure_S7

ciy837_suppl_Supplementary_Figure_S8

ciy837_suppl_Supplementary_Figure_S9

ciy837_suppl_Supplementary_Figure_S10

ciy837_suppl_Supplementary_Figure_S11

ciy837_suppl_Supplementary_Figure_S12

ciy837_suppl_Supplementary_Appendix_A

ciy837_suppl_Supplementary_Appendix_B

## Notes

***Acknowledgments.*** The authors acknowledge Nia Roberts for her help and expertise in developing the search strategy and the reviewers for their helpful and insightful comments. The views expressed are those of the authors and not necessarily those of the National Health Service, the National Institute for Health Research or the UK Department of Health.

***Financial support.*** This work was supported by the National Institute for Health Research (NIHR) School for Primary Care Research (funding round 11, award 309). J. L. is an NIHR In Practice Fellow. C. G. is a Wellcome Trust Doctoral Fellow. J. V., T. A., G. H., and A. V. are supported through the NIHR Community Healthcare MedTech and IVD Co-operative Oxford at Oxford Health Foundation Trust (awardMIC-2016–018).

***Potential conflicts of interest.*** All authors: no reported conflicts of interest. All authors have submitted the ICMJE Form for Disclosure of Potential Conflicts of Interest. Conflicts that the editors consider relevant to the content of the manuscript have been disclosed.
