# Supplementary figures and images for: The Clinical Utility of Point-of-Care Tests for Influenza in Ambulatory Care: A Systematic Review and Meta-analysis
Source: Clin Infect Dis. 2018 Oct 4;69(1):24–33. doi: 10.1093/cid/ciy837 (PMC6579962; doi:10.1093/cid/ciy837)

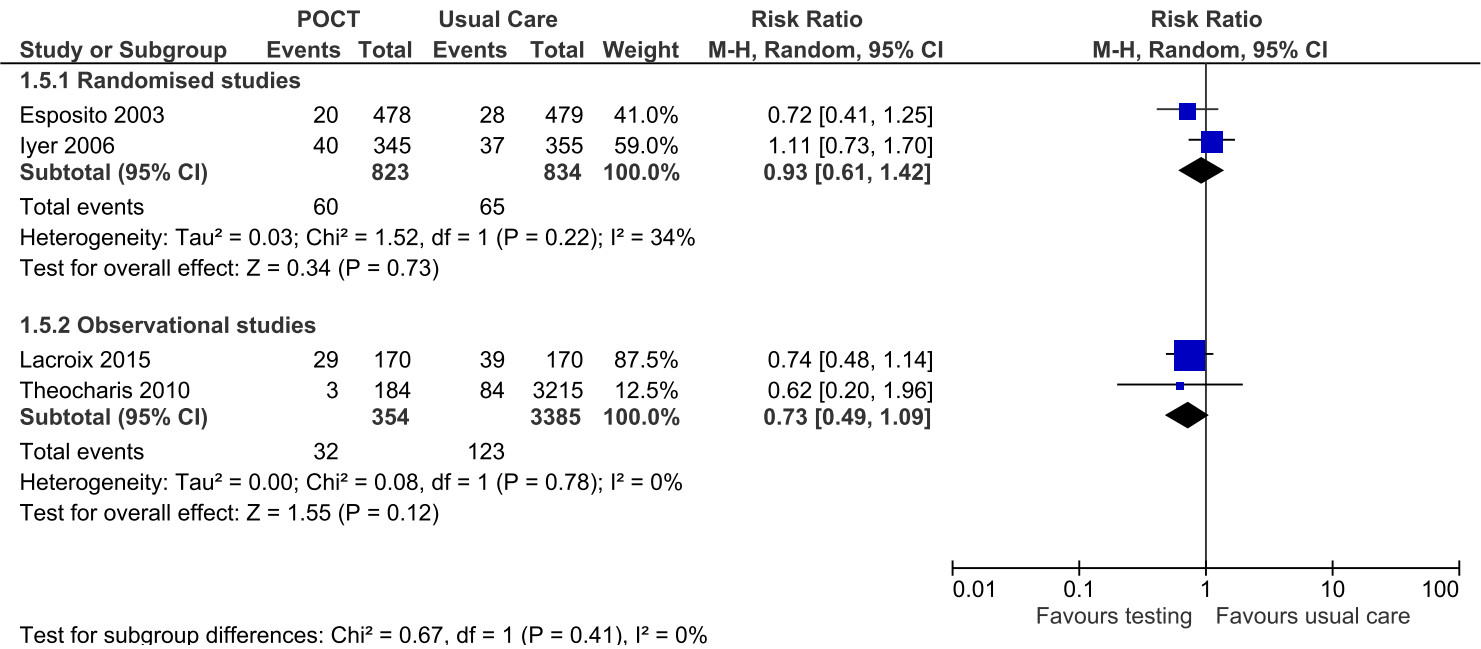

Supplement: ciy837_suppl_Supplementary_Figure_S1 [file ciy837_suppl_supplementary_figure_s1.pdf]

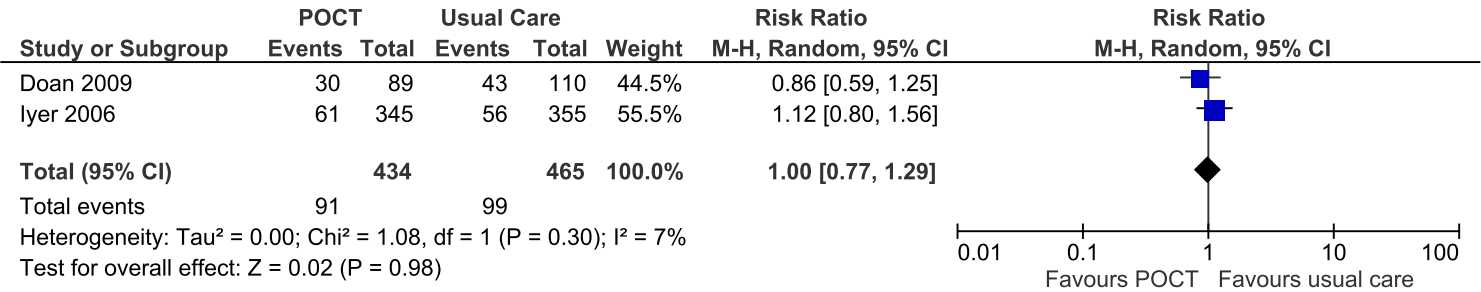

Supplement: ciy837_suppl_Supplementary_Figure_S2 [file ciy837_suppl_supplementary_figure_s2.pdf]

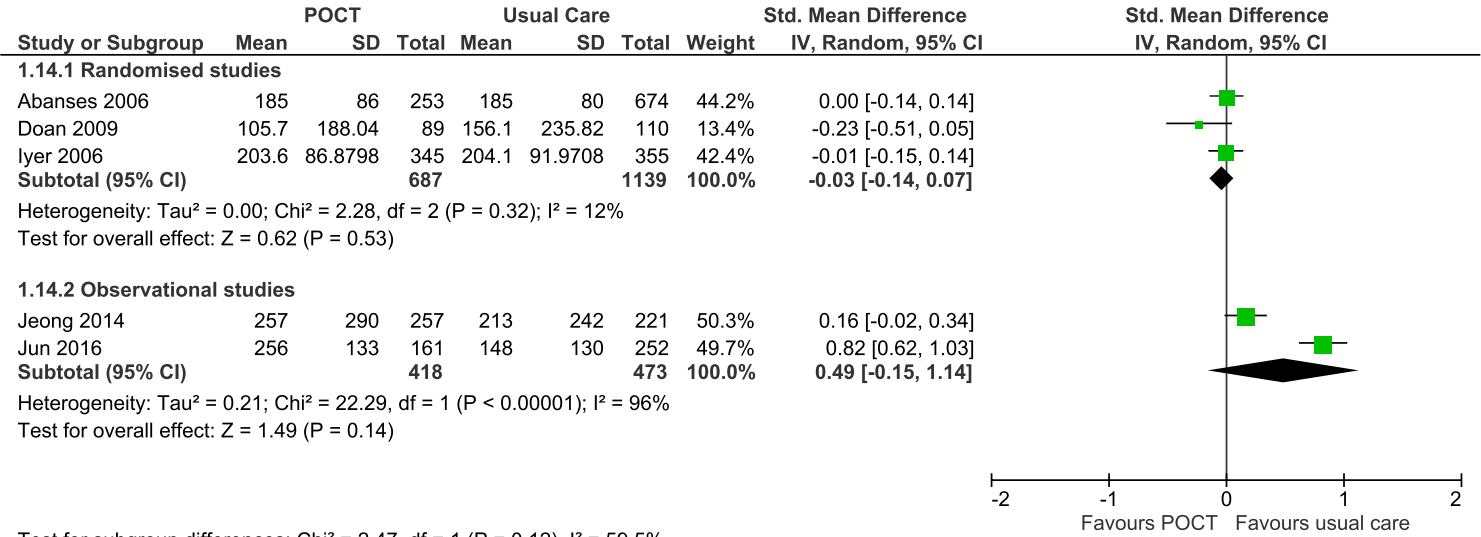

Supplement: ciy837_suppl_Supplementary_Figure_S3 [file ciy837_suppl_supplementary_figure_s3.pdf]

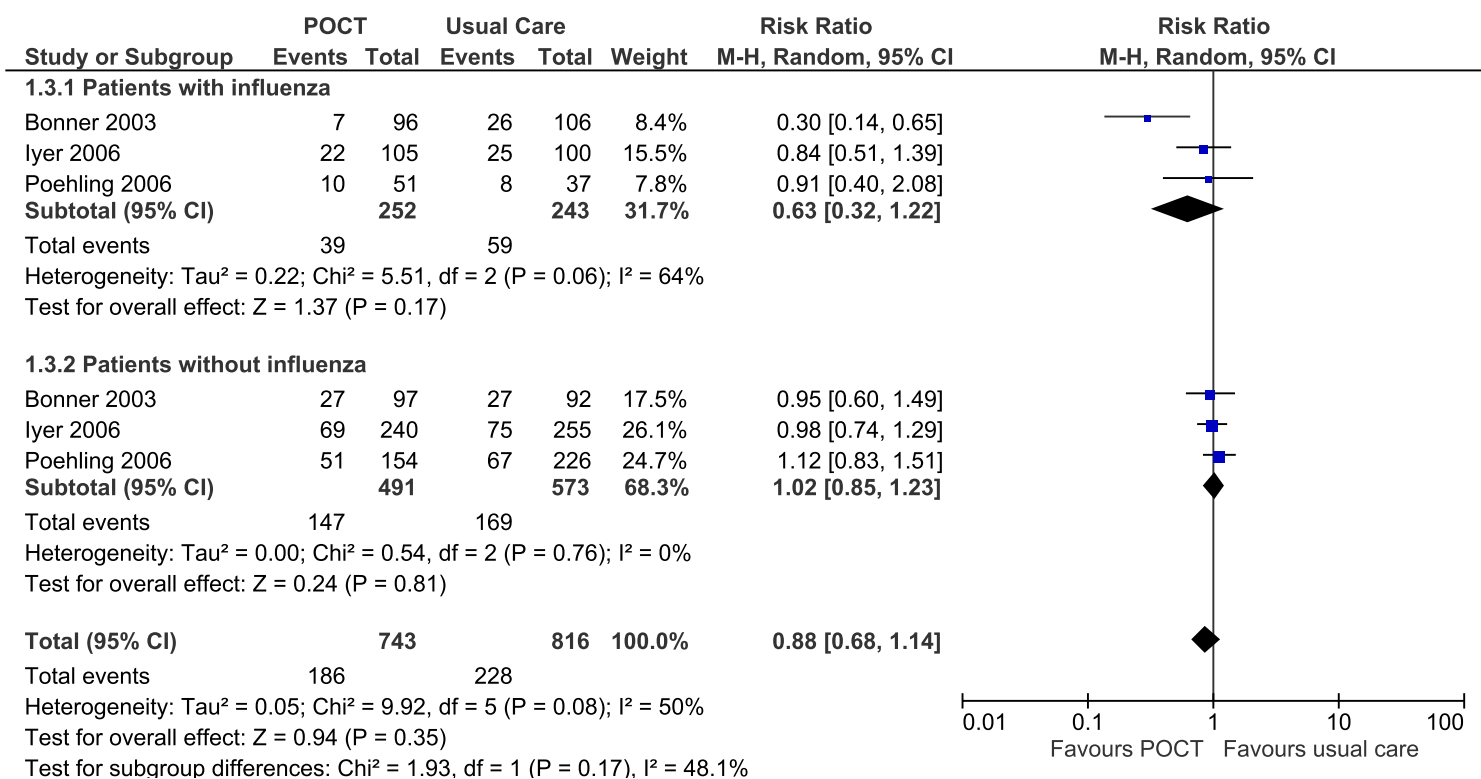

Supplement: ciy837_suppl_Supplementary_Figure_S4 [file ciy837_suppl_supplementary_figure_s4.pdf]

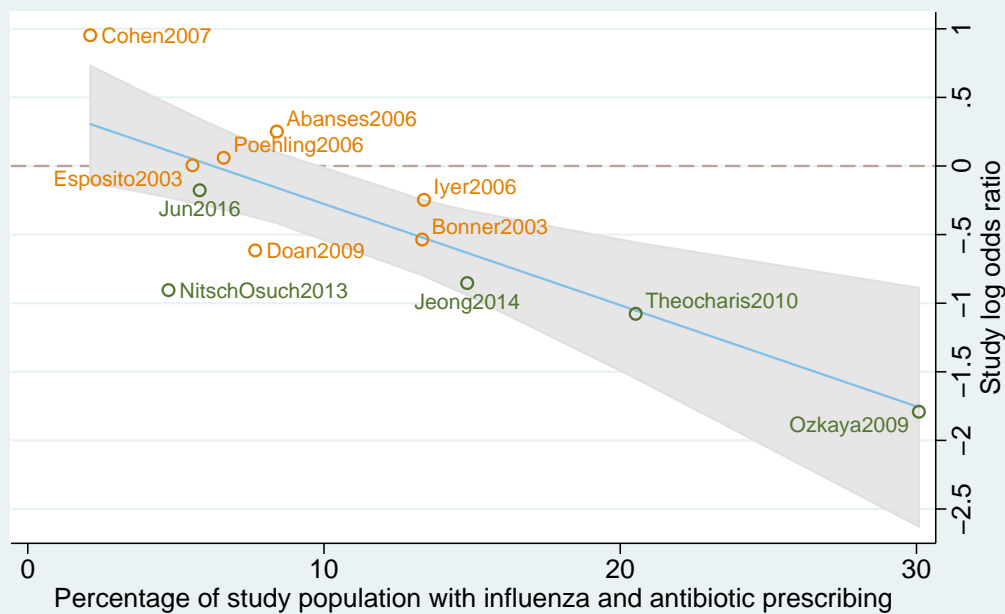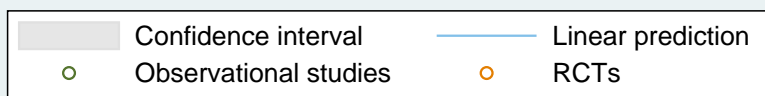

Supplement: ciy837_suppl_Supplementary_Figure_S5 [file ciy837_suppl_supplementary_figure_s5.pdf]

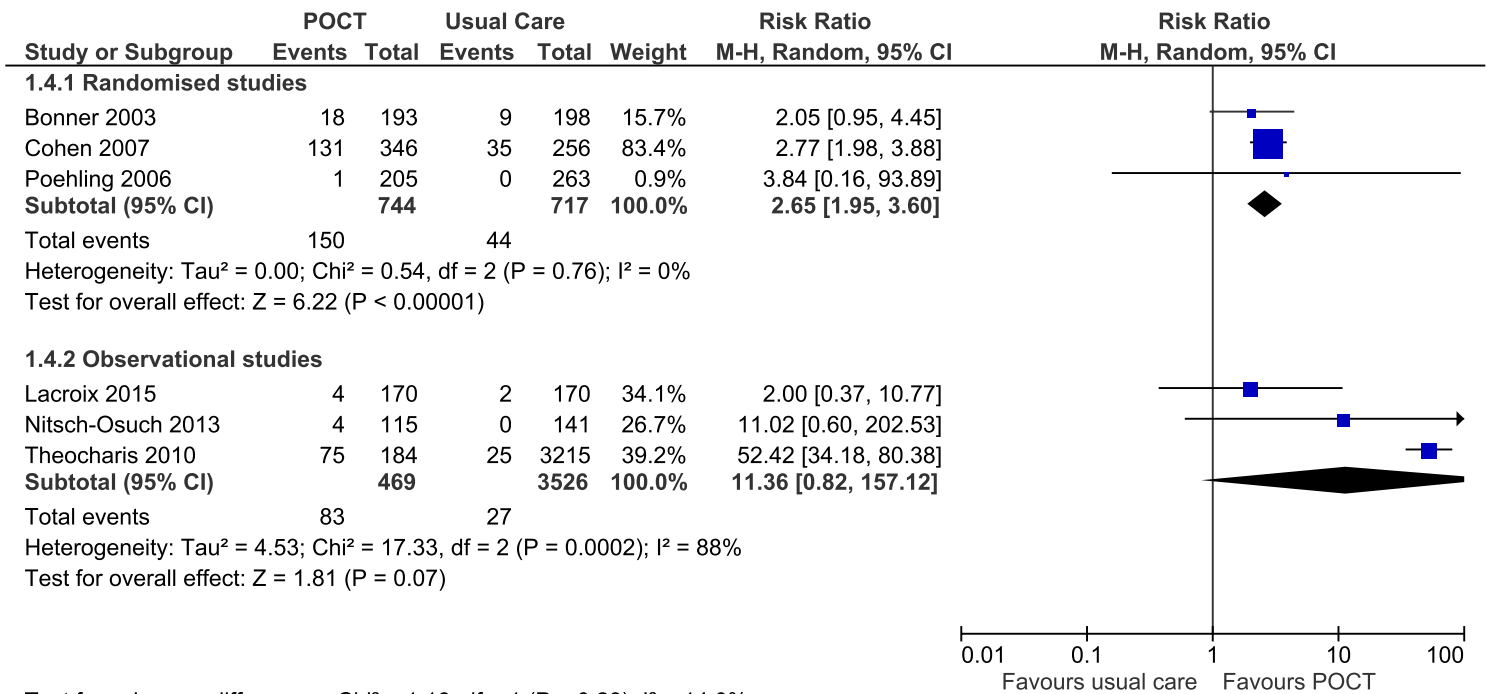

Supplement: ciy837_suppl_Supplementary_Figure_S6 [file ciy837_suppl_supplementary_figure_s6.pdf]

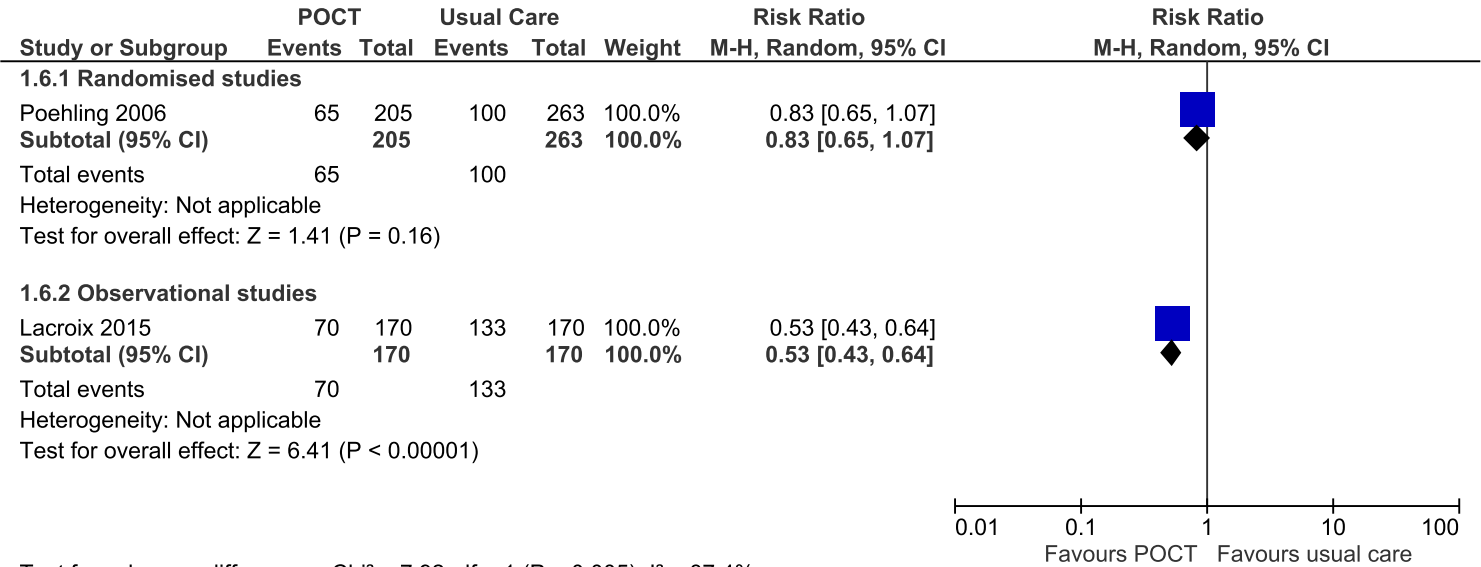

Test for subgroup differences: Chi² = 7.92, df = 1 (P = 0.005), I² = 87.4%

Supplement: ciy837_suppl_Supplementary_Figure_S7 [file ciy837_suppl_supplementary_figure_s7.pdf]

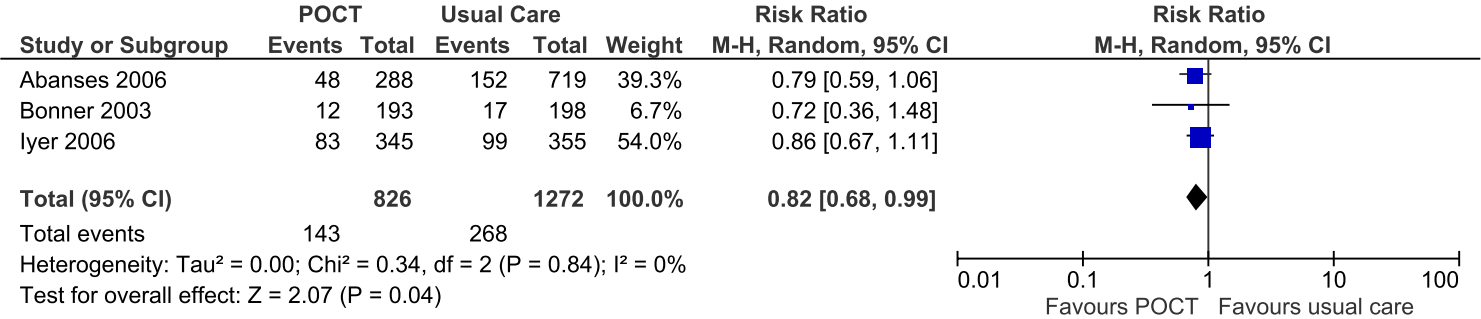

Supplement: ciy837_suppl_Supplementary_Figure_S8 [file ciy837_suppl_supplementary_figure_s8.pdf]

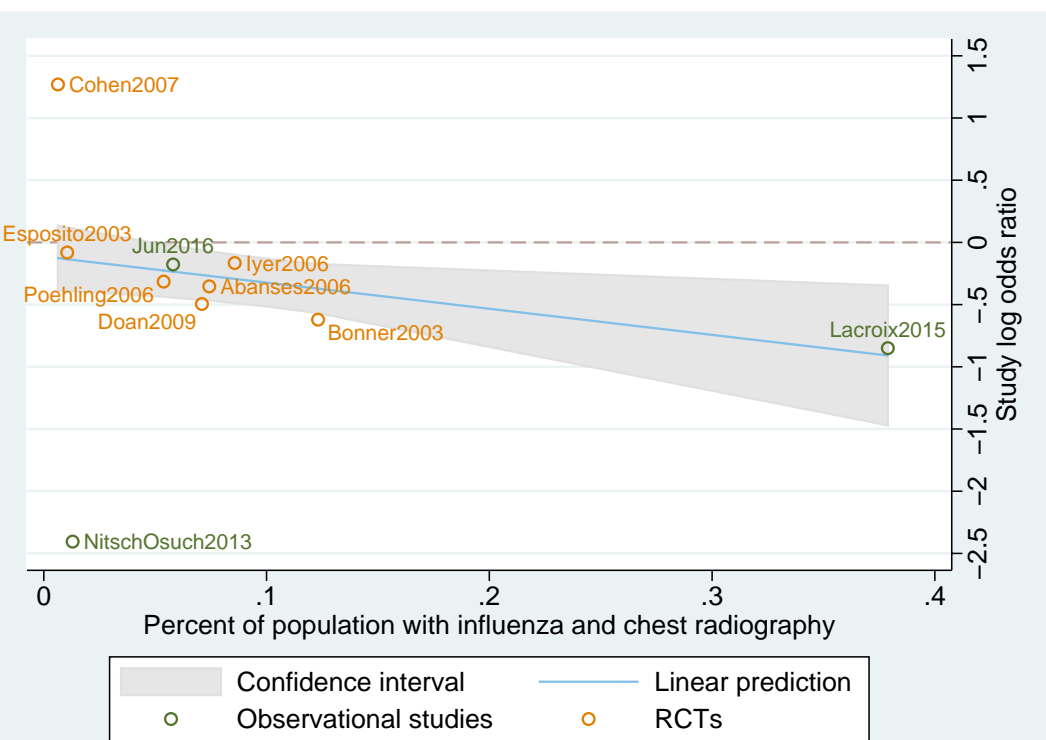

Supplement: ciy837_suppl_Supplementary_Figure_S9 [file ciy837_suppl_supplementary_figure_s9.pdf]

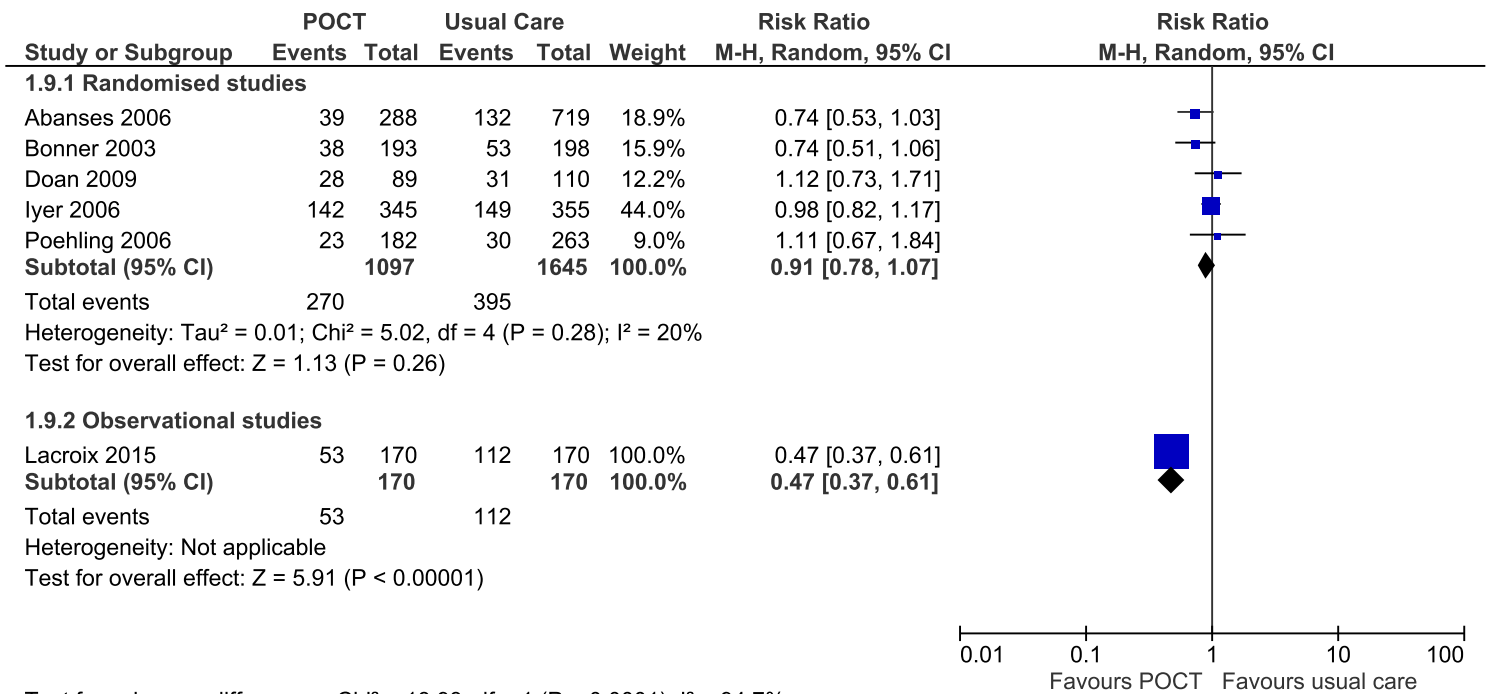

Test for subgroup differences:  $\chi^2 = 18.93$ ,  $df = 1$  ( $P < 0.0001$ ),  $I^2 = 94.7\%$

Supplement: ciy837_suppl_Supplementary_Figure_S10 [file ciy837_suppl_supplementary_figure_s10.pdf]

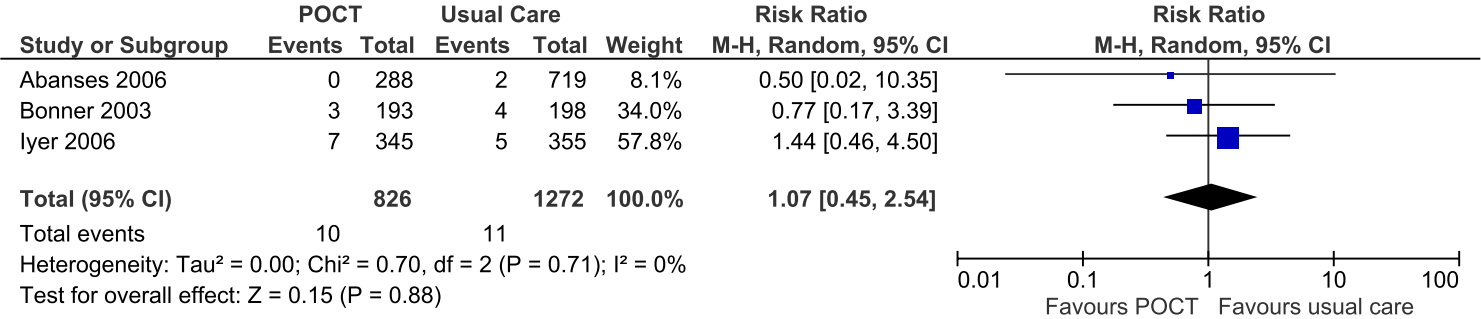

Supplement: ciy837_suppl_Supplementary_Figure_S11 [file ciy837_suppl_supplementary_figure_s11.pdf]

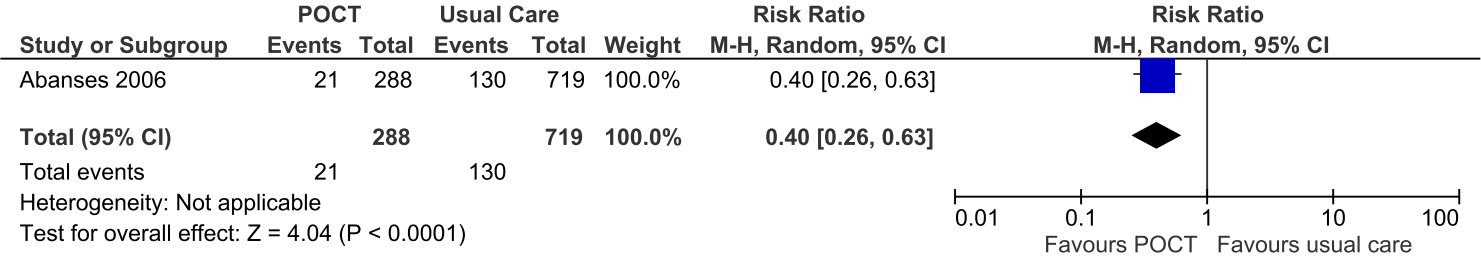

Supplement: ciy837_suppl_Supplementary_Figure_S12 [file ciy837_suppl_supplementary_figure_s12.pdf]
