# Supplementary material for: The Clinical Utility of Point-of-Care Tests for Influenza in Ambulatory Care: A Systematic Review and Meta-analysis
Source: Clin Infect Dis. 2018 Oct 4;69(1):24–33. doi: 10.1093/cid/ciy837 (PMC6579962; doi:10.1093/cid/ciy837)
Supplement: ciy837_suppl_Supplementary_Appendix_A [file ciy837_suppl_supplementary_appendix_a.docx]

**Appendix A : Search Strategy**

| 1 | Ambulatory Care/ |
| --- | --- |
| 2 | exp Ambulatory Care Facilities/ |
| 3 | general practice/ or family practice/ |
| 4 | general practitioners/ or physicians, family/ or physicians, primary care/ |
| 5 | Primary Health Care/ |
| 6 | Office Visits/ |
| 7 | exp Emergency Service, Hospital/ |
| 8 | Emergency Medical Services/ |
| 9 | (ambulatory adj3 (care or setting? or facilit* or ward? or department? or service?)).ti,ab. |
| 10 | ((general or family) adj2 (practi* or physician? or doctor?)).ti,ab. |
| 11 | (primary care or primary health care or primary healthcare).ti,ab. |
| 12 | (emergency adj3 (care or setting? or facilit* or ward? or department? or service?)).ti,ab. |
| 13 | (after hour? or afterhour? or "out of hour?" or ooh).ti,ab. |
| 14 | (clinic? or visit?).ti,ab. |
| 15 | ((health* or medical) adj2 (center? or centre?)).ti,ab. |
| 16 | community health services/ or exp community health nursing/ |
| 17 | Community Health Workers/ |
| 18 | (community adj2 (health or health care or service? or program*)).ti,ab. |
| 19 | (community adj2 (worker? or aide? or volunteer? or assistant? or visitor?)).ti,ab. |
| 20 | ((lay or volunteer) adj2 (health worker? or health aide? or health assistant?)).ti,ab. |
| 21 | ((health* or medical) adj2 (facility or facilities)).ti,ab. |
| 22 | 1 or 2 or 3 or 4 or 5 or 6 or 7 or 8 or 9 or 10 or 11 or 12 or 13 or 14 or 15 |
| 23 | 16 or 17 or 18 or 19 or 20 or 21 |
| 24 | Point-of-Care Systems/ |
| 25 | (("point of care" or POC) adj3 (test* or diagnos*)).ti,ab. |
| 26 | (("point of care" or POC) and (test* or diagnos*)).ti. |
| 27 | poct.ti,ab. |
| 28 | ((rapid or bedside or bed-side or "near patient") adj3 (test* or diagnos*)).ti,ab. |
| 29 | ((rapid or bedside or bed-side or "near patient") and (test* or diagnos*)).ti. |
| 30 | 24 or 25 or 26 or 27 or 28 or 29 |
| 31 | (istat or i-stat or afinion).ti,ab. |
| 32 | 30 or 31 |
| 33 | 22 and 32 |
| 34 | 23 and 32 |
| 35 | 34 not 33 |
