# Supplementary material for: The Clinical Utility of Point-of-Care Tests for Influenza in Ambulatory Care: A Systematic Review and Meta-analysis
Source: Clin Infect Dis. 2018 Oct 4;69(1):24–33. doi: 10.1093/cid/ciy837 (PMC6579962; doi:10.1093/cid/ciy837)
Supplement: ciy837_suppl_Supplementary_Appendix_B [file ciy837_suppl_supplementary_appendix_b.docx]

**Appendix B – Supplementary materials**


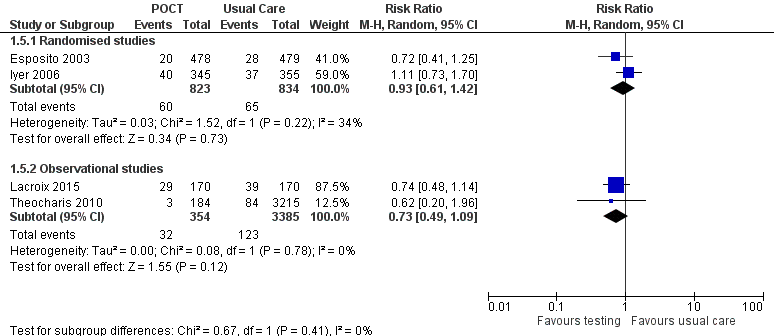


**Figure S1: Hospital admissions**


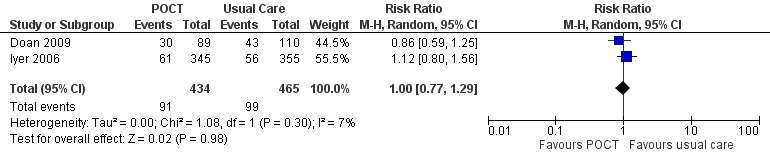


**Figure S2: Return for care**


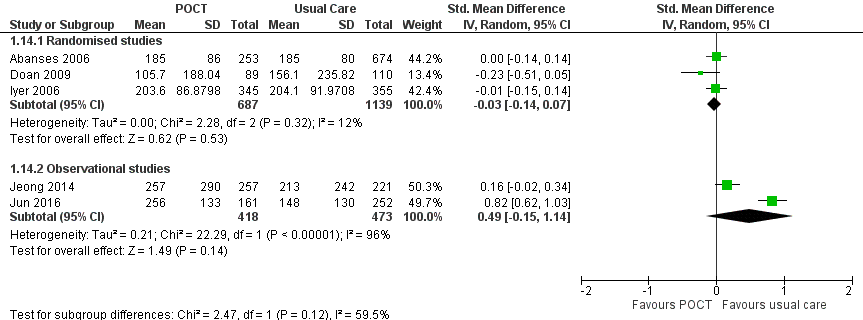


**Figure S3: Time in the emergency department**


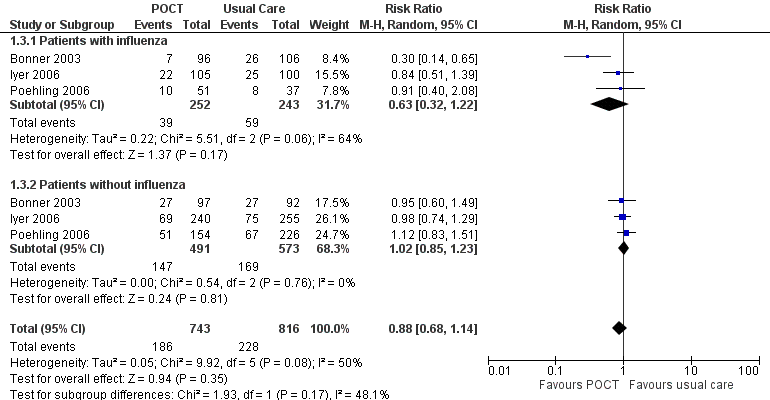


**Figure S4: Antibiotic prescribing by influenza status**


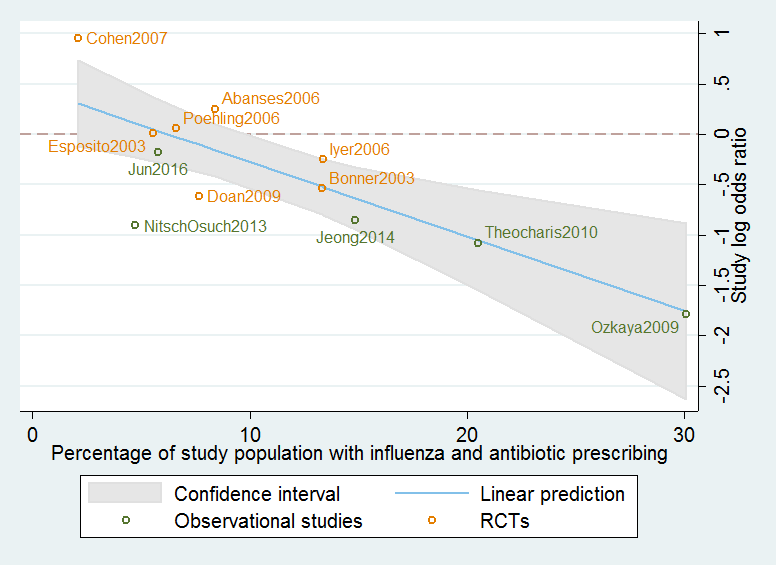


**Figure S5: Meta-regression of study log odds ratio for antibiotic prescribing vs underlying risk of antibiotic prescribing to influenza positive patients (antibiotic prescribing in the control arm multiplied by influenza prevalence)**


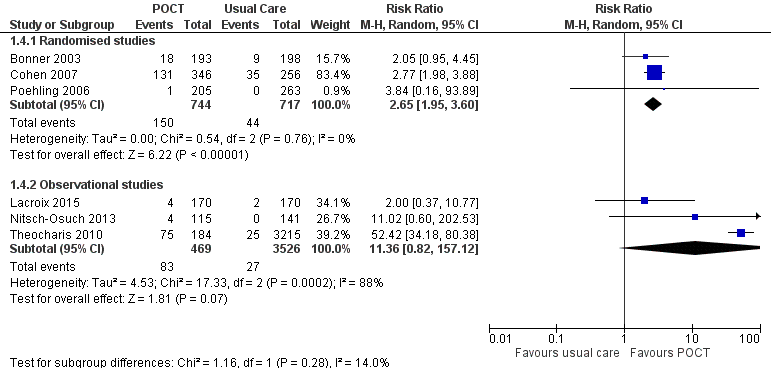


**Figure S6: Antiviral prescribing**


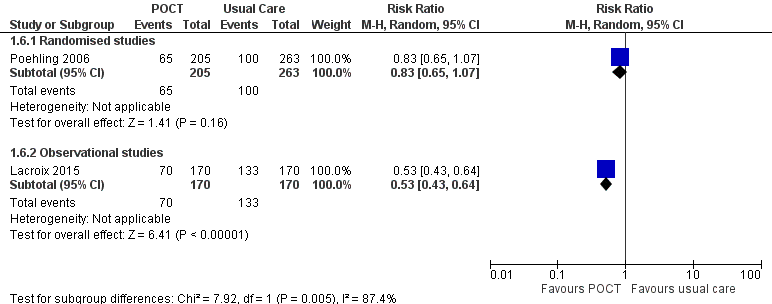


**Figure S7: Composite of any further tests**


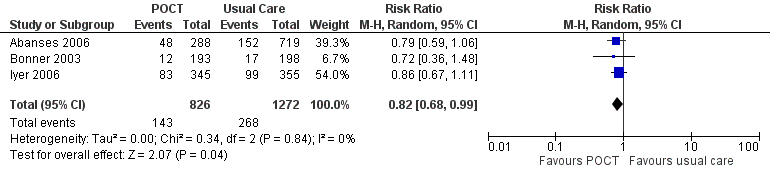


**Figure S8: Blood cultures**


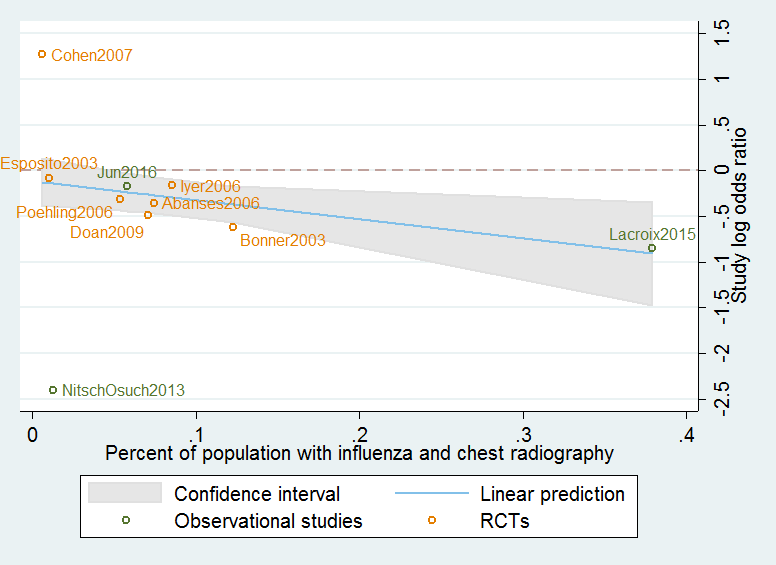


**Figure S9: Meta-regression of study log odds ratio for chest radiography vs underlying risk of chest radiography for influenza positive patients (radiography in the control arm multiplied by influenza prevalence).**


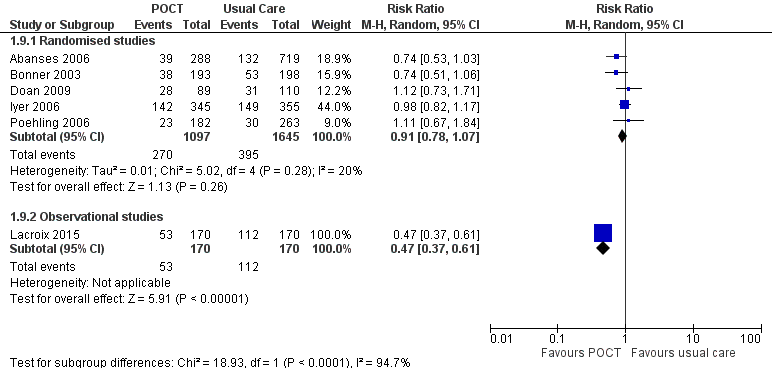


**Figure S10: Urinalysis or urine culture**


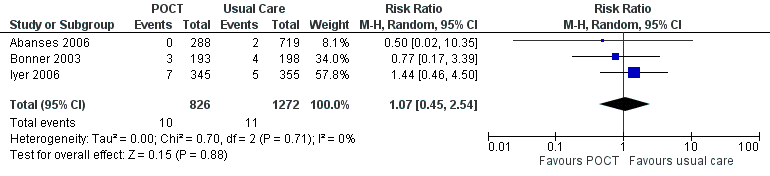
**Figure S11: Lumbar puncture**


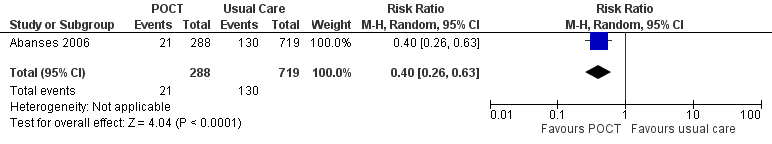


**Figure S12: RSV testing**
